# Model uncertainty, the COVID-19 pandemic, and the science-policy interface

**DOI:** 10.1098/rsos.230803

**Published:** 2024-02-14

**Authors:** Henrik Thorén, Philip Gerlee

**Affiliations:** ^1^ Department of Philosophy, Lund University, Lund 22100, Sweden; ^2^ Mathematical Sciences, Chalmers University of Technology and University of Gothenburg, 412 96 Gothenburg, Sweden

**Keywords:** uncertainty, policy, COVID-19, epidemiology, science-policy interface

## Abstract

The COVID-19 pandemic illustrated many of the challenges with using science to guide planning and policymaking. One such challenge has to do with how to manage, represent and communicate uncertainties in epidemiological models. This is considerably complicated, we argue, by the fact that the models themselves are often instrumental in structuring the involved uncertainties. In this paper we explore how models ‘domesticate’ uncertainties and what this implies for science-for-policy. We analyse three examples of uncertainty domestication in models of COVID-19 and argue that we need to pay more attention to how uncertainties are domesticated in models used for policy support, and the many ways in which uncertainties are domesticated within particular models can fail to fit with the needs and demands of policymakers and planners.

## 1. Introduction

The COVID-19 pandemic has highlighted many of the challenges that arise when science is deployed to support decision-making where risks and uncertainties are rife. In particular, the experience of the pandemic has underscored the dangers, as well as the opportunities, associated with using computational models in this role. On the one hand, computational models have been crucial in structuring, synthesizing, and communicating the scientific understanding—itself constantly changing—of a quickly unfolding situation. On the other hand, computational models are always designed with particular purposes in mind [1], and as such, limited and constrained in many ways. Using outputs from computational models can thus be fraught with dangers and it mandates carefulness and sound judgement, as many have been at pains to point out [2,3].

The unsurprising fact that many models ‘got it wrong’ repeatedly made headline news and sparked sometimes fierce debate [4]. The Imperial College model [5] developed by Neil Ferguson and his team as well as a model developed by the Seattle-based Institute for Health Metrics and Evaluation (IHME)—both influential models—turned out to have wildly exaggerated key outcomes such as daily death rates and hospitalization [3,6]. What struck some critics as particularly egregious was not merely erroneous predictions in a narrow sense, but the misrepresentation and mismanagement of uncertainties and potential (and maybe actual) harms caused by such mismanagement.

In this paper we will discuss computational models of the pandemic and how they have functioned to structure or *domesticate* uncertainties in various ways. The main thesis in this paper is that models are important tools in domesticating uncertainties, and that the precise way in which a model does this matters a great deal for its usefulness in aiding planning and policymaking. Our analysis raises a host of issues, including for example how to manage the trust in science-the-institution [4] and how to appropriately and responsibly communicate scientific knowledge to policy-makers.

## 2. Models and uncertainty domestication

One important role epidemiological models played during the pandemic was to provide predictions and forecasts of how COVID-19 would spread in the future. Such forecasts are always uncertain for a variety of reasons, and the accurate reporting of those uncertainties is often believed to be central in the responsible communication of science to policy makers and the general public, as well as upholding the appropriate division of labour between scientific and decision-making institutions [7]. Hence organizations such as the Intergovernmental Panel on Cimate Change (IPCC) has put considerable time into developing and implementing calibrated uncertainty languages that can be applied across disciplinary contexts (see [8]). This is challenging for all kinds of different reasons, including for instance how different groups (and crucially the intended audience) understand natural language uncertainty terms (e.g. [9,10]), disciplinary differences in how uncertainties are conceptualized (e.g. [11]), what to do when we cannot muster quantitative reports on uncertainties (e.g. [12]), and so on. In this paper, we focus specifically on the role of models and modelling in understanding and shaping uncertainties. That is, models as tools that are themselves involved in the managing and structuring of uncertainties and not merely associated with some degree of uncertainty. The idealizations, simplifications, omissions, and distortions upon which any model is built serve, in part, as a filter for sources of uncertainty, prioritizing among them, highlighting some while ignoring others, and sometimes even acting directly on how uncertainties are represented.

For example, when trying to model an outbreak of COVID-19 in a population, a policymaker might be interested in the impact on the health-care system under various intervention options. In trying to provide relevant forecasts a modeller might focus on some uncertainties, such as for instance what the *R*-value is in the given population and try to account for this inside the model, while ignoring others, such as what happens to the health-care system as it operates close to its operational ceiling for prolonged periods of time, or feedback effects that might occur as a consequence of different non-pharmaceutical interventions. Some of these omitted features may be relevant to the decision situation, but they might nonetheless fall outside a particular model. The point here is simply that choices about *what* gets to be represented within a particular model come to have an effect on what uncertainties that can be managed inside that model.

Moreover, even with respect to processes and parameters that are explicitly represented within a model uncertainty can be managed in different ways. For an uncertain parameter one might settle for a best guess value, a range of values, perhaps with an associated probability distribution, or an extreme value such that the real value is very probably lower or higher. Which option is suitable clearly depends on demands of a given situation but actual choices are in practice often guided by what is convenient from a narrow disciplinary or modelling perspective (cf., [13,14]).

A common way to think about these problems is that the uncertainties involved are particularly problematic, some uncertainties cannot be quantified [15], they are *deep* [12] or *strong* [14] or *fundamental* [16] or *genuine* [17], or there are otherwise intricate second-order uncertainties about the very concepts we use—i.e. uncertainties about which we are uncertain of what kind they are. Beyond the measurable there always seems to linger substantive and difficult-to-know-what-to-do-with uncertainties flowing from e.g. the outcomes of strategic interactions between individuals [18], or fundamental and irreducible uncertainties: the genuinely indeterminate, the fallibility of science, the instability of our concepts, and so on [19]. These kinds of uncertainty, often by definition, fall outside of quantitative uncertainty assessments.^1^

The presence of such uncertainties is significant for several reasons. One is that they make the use of conventional and powerful decision-making frameworks that need probabilistic inputs, such as expected utility theory, impossible to use and therefore (according to some) mandate the development of better decision rules (e.g. [12]). This in turn produces uncertainties with respect to *what* new decision rule to deploy in a given situation—the alternatives are many. Another challenge, highlighted by philosophers of science, is that the presence of uncertainties is an avenue for value judgements to enter into scientific reasoning, for instance by way of so-called inductive risks (e.g. [21–23]). Without delving into the details of these arguments we are presented with a choice. Either we find a way to responsibly integrate value judgements in scientific reasoning and give up the idea of a truly value free science, or we come up with ways of expressing uncertainties in clear ways of communicating uncertainties, perhaps in combination with some good and general principle that can tell us what types or sources of uncertainty we can ignore (e.g. [7]). Regardless of the route one is inclined to take here, considerable challenges arise of both a philosophical and practical nature and hence the uncertainties never quite go away.

For this reason, we prefer to place less emphasis on the *kind* of uncertainty involved, and more on how, in this case our models, structures the uncertainties in a real situation. Here we find Shackley & Wynne's [24] *uncertainty domestication* metaphor apt.^2^ Shackley and Wynne are interested in pointing out how uncertainty can function as a *boundary ordering device* that mediates between groups with partially overlapping and partially diverging interests (scientists and policymakers) and protects the different interests of these actors. Although the point we want to make also revolves around how different actors at the science-policy interface may exchange and use knowledge, our use of this metaphor is somewhat different. To us it conveys the point that in real concrete situations the uncertainties are often overwhelming. In public policymaking and planning we are typically confronted with *wicked problems* [29,30] where it is difficult or impossible to constrain the relevant aspects of the problem situation unambiguously and attempted interventions often have unforeseen (or even unforeseeable) consequences. When scientific models are used to inform planning and decision-making in such situations uncertainties too become difficult to contain. To domesticate uncertainties in this context is parse them, structure them, highlight some and ignore others, in order to make them manageable. This involves trading off and prioritizing between competing values.

A point of comparison might help to illustrate our point. Gärdenfors & Sahlin [31] note that in everyday decision-making situations the decision maker will typically not consider every eventuality when balancing different risks, but rather restrict the set of what is weighed to a smaller set containing not the totality of what could happen as far as they know (what is epistemically possible) but only the decision maker judge to be ‘serious possibilities' [31, p. 369]. The example they provide is that when deciding whether or not to drive, people typically will not go about checking the breaking fluids or if all the wheels are properly attached. This is not because such malfunctions are impossible or would not be dangerous. Rather they are not considered to be among the *serious possibilities*. This restriction of risks makes the decision situation less computationally taxing for the decision-maker.

This is an example of uncertainty domestication. A choice is made with respect to what eventualities and sources of uncertainty to account for, such that some other aim is made easier to achieve—in this case usually then computability. However, when scientific models are used to aid policymakers, the situation is much more complicated. First, although a model certainly involves a choice with respect to which parameters and processes are explicitly represented and which ones are not—and hence what outcomes can be represented—this is not merely a probability threshold akin to what Gärdenfors and Sahlin have in mind where low probability outcomes are disregarded (although that may be happening too). Instead, a range of other considerations may come into play on part of the modeller when uncertainties are domesticated. These include, but are not limited to, the following:

— specific parameters or processes may be perceived to be outside of the domain of expertise of the modeller;
— the uncertainties associated with a specific process or parameter may be perceived to be deep and therefore destabilize the model if they were to be included; and
— the model or modelling framework may not accommodate the process or parameter in question, or it may be uncertain *how* such an accommodation should be achieved.

The point is that some of these motivations may hinge on the perceived scope of a particular discipline or the affordances of some specific model, and so on. In other words, what drives uncertainty domestication in a model or modelling approach need not be informed by the decision situation *per se*, but rather by the constraints and affordances of the model and what is convenient given some set of interests, values, and priorities.

A second point is that since the modelling and decision-making tasks are distributed among different actors with different roles there is always the potential for divergences, gaps, and conflicts among these actors with respect to their interests, values, and priorities (c.f., [32]). The modeller may prioritize stabilizing their model, which may involve disregarding some sources of uncertainty, whereas the policymaker may be guided by other considerations, such as for instance been accountable for preventing certain outcomes, which may lead to other choices being made with respect to which sources of uncertainty to focus on.

To conclude this sub-section, the point is not that uncertainty domestication in the sense that we use it is somehow in itself problematic. As we understand it, it is for the most part a prerequisite of modelling in the first place. In the Discussion section we will return to the issue of what amounts to responsible uncertainty domestication in models used to support decision-making.

We will now proceed with three examples that highlight the role and consequences of uncertainty domestication in computational models of COVID-19.

## 3. Uncertainty in epidemiological models of COVID-19

The handling of uncertainty has had important consequences for the modelling of the COVID-19 pandemic, both in terms of influencing model structure and how uncertainty in underlying assumptions propagate through the model and into model predictions. In the following, we present three examples of how domestication has the potential to impact model structure and in turn the communication of uncertainty to stakeholders.

### 3.1. Case 1: the same data—different models

To set the scene, we need to discuss how model structure and in particular the complexity of the model affects its ability to describe real-world data.

One dimension along which we can characterize model structure is in terms of *rigidity*. By this we mean the span of possible dynamics a model can exhibit. Can the model only produce a constant increase (e.g. exponential growth), a single-peaked curve (e.g. such as the compartmental susceptible-infectious-recovered (SIR)-model) or more complex temporal dynamics with multiple peaks? A model that can only exhibit a narrow band of dynamics is more *rigid* than one that can exhibit a broader band of dynamics.

This is often related to the number of parameters or degrees of freedom in the model. A comparison with statistical models for linear regression can be helpful. For example, a linear regression model (of the form *y* = *ax* + *b*) can only produce a linear relationship between input *x* and output *y*. What we can adjust to fit the model to the data is the intercept (*b*) and the slope of the line (*a*). A quadratic regression model (of the form *y* = *c* + *dx* + *ex*^2^) can describe both a linear relationship (by setting *e* = 0) and a purely quadratic relation (by setting *d* = 0) between input and output (and a combination thereof). Here the linear model (with two parameters) is more rigid than the quadratic model (with three parameters) since the latter can describe a wider range of relationships in the data.

Returning to the computational models of COVID-19 transmission, the same type of reasoning can be applied. Models with a small number of parameters are more constrained and thus more rigid compared to larger more complex models in terms of possible trajectories. Here, we typically find statistical models in the more rigid end of the spectrum, agent-based models at the other extreme and compartmental models somewhere in between.

What bearing does model rigidity have on uncertainties in model predictions? To answer this question, let us consider two models, one more rigid than the other, that are calibrated against the same time series. If one of the models indeed was the true one (e.g. the time series was generated synthetically from the model) we would expect our parameter inference method to provide narrower confidence and/or prediction intervals for the correct model independent of model rigidity.

However, in real-world scenarios of disease transmission the time series was generated by a process which is infinitely more complex than any computational model. In this case both models are crude simplifications (as all models are) and uncertainties in the estimates of parameter values (which propagate to uncertainties in predictions) are to a large extent driven by model rigidity. Simply put, it is easier to pin down a smaller number of parameter values with the same number of data points. This is most easily seen in linear regression where, assuming the exact same deviation between model and data, a model with a larger number of parameters exhibits wider confidence intervals for the estimated parameters and corresponding prediction intervals. This typically holds true also for the nonlinear regression models such as the IHME-model, which was used by the White House in early 2020 [33].

This observation points to the fact that confidence intervals are always conditioned on the choice of model and only display uncertainty conditioned on model choice. They are *model-based uncertainties* (cf., [13]). The confidence intervals represent some set of uncertainties that remain once we have disregarded some other set of uncertainties, such as for example structural uncertainties, problems with the data, and so on. These assumptions domesticate uncertainties in the sense that whatever remains within the purview of the model can be captured in precise ways as statistical confidence intervals. Often, however, they do not capture the uncertainties pertaining to the real situation.

**Figure 1.**
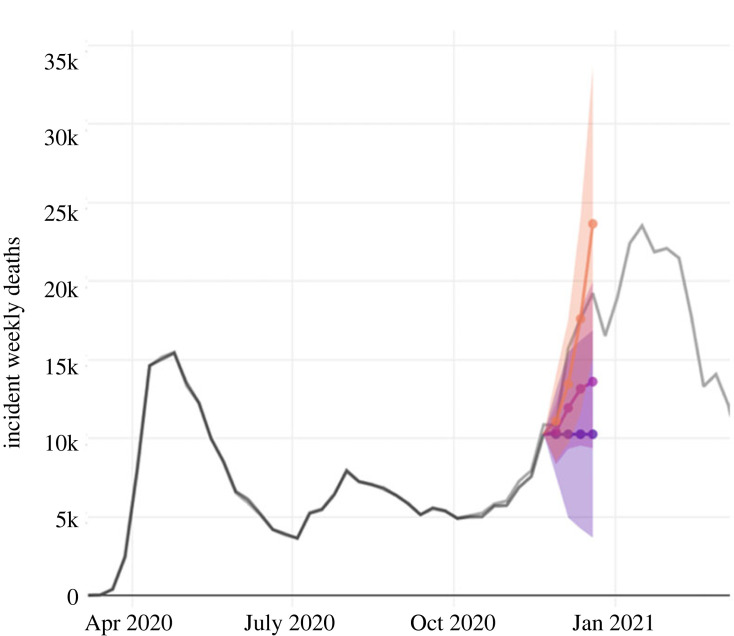
Forecast from the COVID-19 Forecast Hub which is maintained by the CDC. The three models shown are the COVIDhub-baseline (blue), Columbia_UNC-SurvCon (orange) and CU-select (purple). Figure adapted from https://viz.covid19forecasthub.org/.

The fact that prediction intervals depend on model choice can readily be seen in the forecasts of the COVID-19 pandemic presented by the United States Centre for Disease Control (CDC; figure 1). They have aggregated model predictions submitted by research groups and individuals, that have all been trained on the exact same data. The models make predictions with varying degrees of certainty and the variation is solely owing to different modelling assumptions.

### 3.2. Case 2: evolution of novel virus variants

The first wave of the pandemic that occurred during the spring of 2020 was driven by the original strain of SARS-CoV-2, later dubbed the Wuhan variant. The possibility of evolution of the virus and the emergence of novel variants was raised early in the pandemic [34], and novel variants with increased transmissibility and the ability to escape acquired immunity posed a serious challenge to efforts that aimed at containing the spread of the disease. This threat was indeed realized with the emergence of the alpha variant in England during November 2020. This variant showed a 30% increase in transmissibility and also increased the risk of severe disease and hospitalization [35]. The alpha variant spread globally and resulted in secondary waves in several countries. It was later outcompeted by the even more transmissible delta variant during early 2021, which in turn was replaced by the even more transmissible gamma variant in mid-2021. The latest variant to appear and dominate disease transmission on a global scale is the omicron variant, which is notable for its ability to escape the immune response of individuals that have been previously infected by other variants and also fully vaccinated individuals.

All viruses are known to evolve, but what is particularly striking about the evolution of SARS-CoV-2 is the high rate at which novel variants spread across the world. For omicron the time from its detection in South Africa until it had established itself in a majority of the world's nations was approximately six weeks. This short time frame poses severe challenges to attempts at predicting the course of the pandemic on a national or regional scale since model predictions are typically based on present variants.

In principle it would be possible to account for the emergence of novel variants in pandemic models, but in practice we are limited both by our knowledge of virus evolution and the uncertainty induced by the stochastic nature of evolution. In other words, if we had perfect knowledge about the properties of *all possible* variants of SARS-CoV-2 and included this information into a global model of disease transmission, then we could at least in principle describe the evolutionary dynamics of SARS-CoV-2. However, the space of possible variants is extremely large, and the dynamics dominated by stochastic effects, implying that such a model could at best provide a probabilistic view of possible outcomes, with massive uncertainties associated with specific predictions. One suspects that trying to manage uncertainties in this way runs a serious risk of undermining such a model's usefulness in many decision-making contexts.

However, this does not imply that mathematical and statistical models cannot be used when studying the evolution of SARS-CoV-2, but such tools have to be used with care and can instead provide hints about possible future trajectories of the pandemic. One such example is the work by Day *et al*. [34] in which they carry out a mathematical analysis of a two-strain dynamical model of disease transmission of COVID-19. This type of analysis can reveal which traits of the virus (e.g. transmissibility, immune evasion, fraction of asymptomatic cases, disease severity) are under positive and negative selection. Since novel mutants are produced continuously as the disease spreads, this analysis can give hints as to which traits we can expect to see in novel variants that displace previous variants. Another example where statistical modelling can inform us on the dynamics of novel variants is the work by Obermeyer *et al*. [36]. By analysing 6.4 million SARS-CoV-2 genomes and comparing it to the growth in cases at different regions, they created a statistical model that can predict the growth rate of a novel variant based solely on its genome sequence. The prospective accuracy of the model is yet to be determined, but it highlights how modelling can be used to obtain information on novel variants during the early stages of growth.

In summary the emergence of novel variants creates vast uncertainties even on fairly short time scales. Modellers typically domesticate these uncertainties by placing them outside of models used for short-term predictions (on the order of months). The impact of novel variants in the longer term can, as mentioned above, improve our understanding of virus evolution, but such models can only provide partial clues about the future of the pandemic.

### 3.3. Case 3: theoretical incongruence

We will now discuss an example of COVID-19 modelling which highlights the importance of domestication of uncertainty and the consequences of limited domestication.

Following the impact and media coverage of the Imperial model, which has been claimed to have been the major reason for imposing a strict lockdown in the UK in March 2020, many similar models were constructed during the spring of 2020. One such example is a model by Kamerlin *et al*. which aims at describing the development of the COVID-19 outbreak in Sweden [37]. Like the Imperial model it is an agent-based model (ABM) where individuals are represented as agents within a computational framework, which specifies how the agents behave and interact with one another. Such models, also termed micro-simulation models, have been used previously in epidemiology as well as in other fields such as economy, sociology and ecology.

ABMs typically aim at a more realistic description of a phenomena compared to other dynamical models such as compartmental models. This implies that they contain a large number of parameters that describe how the agents behave and interact. The sheer number of parameters make it difficult if not impossible to estimate parameter values solely from historical data. In terms of an epidemiological model, the data at hand consists of the number of hospital admissions, cases and deaths whereas the model contains on the order of hundreds of parameters. Fitting all model parameters to data is not an option since the uncertainty (and lack of uniqueness) of the parameter values would render the model useless. Instead, one relies on a combination of parameter values obtained from the literature (e.g. the Imperial model) and estimating a small subset of the parameter using historical data.

The Kamerlin model strongly over-predicted the severity of the pandemic. Model simulations showed that if the mitigation measures that were imposed at the time of publication in early April 2020 were to continue, the death toll in Sweden would reach 90 000 by the 1 June 2020. In reality the death toll at the end of May (without any major changes to public restrictions occurring since the publication) reached approximately 6000, an order of magnitude less than predicted.

There are several possible explanations for the failure of the model to capture the dynamics of the pandemic, and we will here explore a single cause related to domestication of uncertainty. ABMs have the capacity to capture detailed information about individual behaviour and societal structure. This makes it tempting to include every aspect of disease transmission. However, this is associated with an increase in model complexity. This growth in complexity not only entails a larger number of uncertain parameters, but more worrisome is that the sub-models created are associated with vastly different fields of research with specific domain knowledge. The authors of the Kamerlin model have included knowledge from and made simplifying assumptions related to the following fields of research:

— **demography:** the age-distribution and household size distribution of Sweden is included since it affects disease transmission;
— **epidemiology:** the model describes how the disease spreads, its incubation time, infectiousness etc.;
— **population geography:** the model accounts for where people live, work and go to school and also how they travel;
— **sociology:** assumptions are made about the compliance to lockdown policies and how people react to reported deaths;
— **economic geography:** the model includes statistics on the size and location of workplaces and hospitals;
— **statistics of education:** the model includes data on the size and location of schools and universities;
— **medicine:** the model has to account for how people are affected by the disease, the hospitalization rates, intensive care unit (ICU) rates, death rates etc.; and
— **hospital logistics:** in the model, assumptions are made about ICU capacity, ICU admission criteria and duration of treatment.

It should be clear that no single research group can make confident modelling assumptions in all the above fields of research, and even in the context of an interdisciplinary effort this type of model would take a very long time to construct. While claiming to model the pandemic with a high level of realism, what actually occurs is that by accounting for a large number of societal and medical processes, which are poorly understood and hence associated with large parametric uncertainty, increasing levels of uncertainty are introduced into the model.

## 4. Discussion

The above examples illustrate well how modelling choices and strategies are intertwined with how uncertainty comes to be domesticated. Although uncertainty domestication can sometimes involve substantive commitments pertaining to e.g. what uncertainties *are*, often the practice of uncertainty domestication, as far as models are concerned anyway, is connected to the idealizations, omissions, distortions, simplifications that modelling inevitably involves and is a major reason why we find models useful to begin with [1,38,39].

As we understand uncertainty domestication generally it can be both what motivates specific idealizations and omissions, and a side-effect of, for instance, idealizing in certain way. As pointed out above, sometimes a modeller is looking to control and delimit the influence of some sources of uncertainty on a model, for instance by ignoring or excluding these sources of uncertainty altogether, or by constraining the domain of applicability of the model. Here, domesticating uncertainties drives modelling choices directly in the sense that the very point is to limit the influence of ‘problematic’ uncertainties on a specific model. This can be important in particular in forecasting exercises when one is explicitly trying to say something about the future state of a system, such as the case of virus evolution discussed above. The purpose of domesticating uncertainties in such situations can be to, for instance, produce tidier forecasts or projections, to avoid destabilizing a model or modelling framework, or isolate specific sources of uncertainty (and be precise about them).

Domestication of uncertainty can also figure indirectly as a consequence of choices made for other reasons, such as limitations imposed by certain modelling frameworks. The case of model rigidity and its influence on confidence intervals is one such example.

The norms and conventions that guide what is admissible to do in the face of different kinds or sources of uncertainty is tightly connected to specific disciplines or fields. For example, climate scientists do not attempt to themselves factor in human responses to climate change in their models. Partially, it would appear, they believe this to be the domain of other disciplines, and this problem has indeed been tackled by climate economists using integrated assessment models. The epidemiologist that disregards how the healthcare system itself may adapt to or be disrupted by a pandemic outbreak might equally maintain that this outside of her domain of expertise, although it may be important to policy-makers and other end-users of model projections. Attempting to expand a model here may trigger having to confront difficult to deal with uncertainties. For example, uncertainties concerning how to model a certain process within a specific modelling framework.

The point is that norms and conventions concerning the appropriate way of handling specific sources of uncertainty, which ones we can readily avoid and which ones that have to be handled with care, differs between disciplines and depends, at least in part, on what is central and important in that discipline [40,41]. As pointed out in §2, challenges arise at intersections between communities embracing different ideas about what is central and important, such as when uncertainty domestication is carried out in one context, that is governed by one set of values, norms, and conventions, and the resulting model results are deployed in another context, where other norms and conventions reign and other judgements are made regarding what is important. An example of this was seen with the Kamerlin model where researchers from other disciplines than epidemiology constructed a model which was of questionable value from an epidemiological perspective and difficult to assess for a presumptive policymaker if they were rely on it. When such value conflicts remain unacknowledged or poorly understood, miscommunication is a grave risk.

In such situations where there is a lack of coordination between scientific knowledge providers and end-users, the knowledge can become either easily misunderstood, or it simply fails to get any traction in the context of application. Epidemiological models that are pressed into service informing policy and planning, presents such a risky situation. Here the models may be developed with an eye towards what epidemiologists or modellers think are important and interesting, and their perception of what constitutes their domain of expertise, whereas planners and policy-makers operate on other criteria, for example a certain set of responsibilities such as managing public health, the fulfilment of which they can be held accountable for.

At this junction a clarification is in order. There is of course nothing that forces modellers to deploy *only* conventional measures of uncertainty (e.g. confidence/prediction intervals or credible intervals in the case of Bayesian modelling), in their communication with decision-makers, but the practice has been common during the pandemic [42]. One alternative would be to take a more holistic approach to uncertainties where model dependent measures are complemented with e.g. (subjective) confidences, as is done by the IPCC [8,9,43]. However, this is probably no panacea either. Instead, what we should focus on, is how ways of domesticating uncertainties within models *fits* with the constraints and demands of a specific decision-making or planning situation. Here we are inspired by e.g. Wendy Parker [1] and Eric Winsberg [39], that argue the model evaluation generally has to be carried out in accordance with their *fitness-for-purpose*, i.e. we use models towards specific ends (prediction, projection, explanation, supporting decisions, etc.) and whether they are good or not depends on how well they serve those ends. The idea of uncertainty domestication and fit can be understood within such an overarching framework.

This then needs to interface with a number of features specific to the decision-making situation. Let us think of a presumptive decision-maker not as a generalized optimizer, but rather as having a specific place and role within some institutional structure. Suppose this place and role within this structure determines (among other things) what her precise responsibilities are and what she can be held accountable for. Moreover, the decision-maker is likely to have some expertise, maybe even modelling expertise, which may inform her in interpreting model derived knowledge claims. Then it seems that models deployed to inform her should domesticate uncertainties such that it fits her specific responsibilities, in light of her ability to digest model derived knowledge, and in compliance with some more general framework for how division of labour and responsibilities between experts and decision-makers is to be handled in the specific situation.

When framing the issue in terms of fit we are drawing attention to how the way a model (or some other scientific representation) domesticates uncertainties, interfaces with these (and perhaps other) aspects of a decision situation. The suggestion that attention to model fit should impact the domestication of uncertainty opens many ways to construct functional science-policy interfaces. It also helps us identify ways in which coordination failures in terms of model fit may hamper knowledge exchange between experts and policymakers. Let's consider two options.

Suppose a model is used to inform a decision-making process and the way uncertainties are domesticated within that model exactly fits what is relevant to that decision-making situation. In such situations it seems that the model derived knowledge can be directly implemented to drive decision-making. Here the decision-makers may be highly ignorant of details in the models, perhaps with the understanding that the models do indeed fit their needs to begin with. This might be because modellers are accountable for errors, but perhaps more likely because there are conventionalized ways of integrating model derived knowledge in the decision-processes, such that individual decision-makers do not need to themselves assess the models.

In other situations, we might rely on the decision-maker to be more actively involved in closing the gap between how models domesticate uncertainties and what is relevant in the decision-making situation. The models might ignore sources of uncertainty that the decision-maker needs to take into consideration. In the context of COVID-19 for instance, various potential social costs and risks with different interventions were usually not part of the modelling, and thus something that was up to the decision-makers to consider [3]. This of course also involves managing and evaluating uncertainties pertaining to those costs and risks. This potentially requires more on part of the decision-maker. Notably, supposing that it is possible for the decision-maker to carry out this operation of fitting model derived knowledge to the decision-situation, this can be preferable.

The main risks we see have to do with coordination coming apart. If model outputs are produced outside of the context in which the decisions are made, this is vastly more taxing for the decision-makers to digest. This in turn may lead to model outputs being practically meaningless to end-users, impossible to implement in practice, or otherwise to severe misunderstandings of how the models should be deployed in a given decision-making situation. Downstream effects of such misunderstandings are first and foremost poor decision-making, but it also raises issues of accountability and can lead to situations where uncertainties arise concerning who is to be held responsible for error or mismanagement.

There are several concrete steps one may take to mitigate such risks. The above discussion points towards fitting how uncertainties are domesticated to the specific conditions, constraints, and affordances that are salient in a given decision making or planning situation. This may in turn be achieved in any number of ways, but co-production of knowledge already at the modelling phase strikes us as a promising way of encouraging and facilitating closer interactions between policy-makers and modellers. This is likely to be important in evolving situations where some of the relevant features of the decision situation are likely change over time.

It is also worthwhile to consider uncertainty domestication and fit in relation to transparency as transparency has often been seen as a crucial in managing uncertainties (or managing values) (e.g. [7,22]; see also [44]). To us it seems that one point the uncertainty domestication concept illustrates is that transparency about uncertainties, at least in a narrow sense, may be neither sufficient nor necessary for effective science-for-policy. It is not sufficient in the sense that merely attaching uncertainty reports to e.g. model derived knowledge claims can obscure as much as it reveals if it is unclear what kind of uncertainty domestication that underpin the uncertainty reports. Concerning necessity, arguably, if fit can be achieved, then transparency is less crucial (although other factors such as trust are bound to come to the fore).

To conclude this section, we are hardly the first to highlight the need for deeper coordination between the providers of model-based knowledge and the end-users of that knowledge, but it is a lesson worth repeating. The experiences of the pandemic clearly illustrate this point.

## 5. Concluding remarks

Our purpose in this paper has not been to relativise the treatment of uncertainty in modelling in order to throw doubt over modelling results. The point of using the idea of uncertainty domestication is to draw attention to the precise ways in which structuring uncertainty within specific models interfaces with features of the decision situations that the models (or their outputs) are supposed to inform. Applying this perspective to the science-policy interface highlights the need for models that fit specific decision contexts and draws attention to processes that facilitate exchanges where diverging interests and priorities between different actors with different roles may lead to coordination failures.

## Data Availability

This article has no additional data.

## Footnotes

1 We are of course not without options here, e.g. Betz [7] and Hansson [20]. Moreover, on a Bayesian approach where all uncertainties can be thought of as degrees of belief some of these challenges are dissolved—but then again, uncertainties remain as to whether this perspective is actually the right one.

2 The metaphor is quite common, although typically not used in the precise way we use it here. See [25–27]. See also [28].
